# The contrast sensitivity function of the praying mantis *Sphodromantis lineola*

**DOI:** 10.1007/s00359-015-1008-5

**Published:** 2015-04-18

**Authors:** Vivek Nityananda, Ghaith Tarawneh, Lisa Jones, Natalie Busby, William Herbert, Robert Davies, Jenny C. A. Read

**Affiliations:** Institute of Neuroscience, Henry Wellcome Building for Neuroecology, Newcastle University, Framlington Place, Newcastle upon Tyne, NE2 4HH UK

**Keywords:** Praying mantis, Contrast sensitivity, Visual ecology, Separable tuning, Optomotor

## Abstract

The detection of visual motion and its direction is a fundamental task faced by several visual systems. The motion detection system of insects has been widely studied with the majority of studies focussing on flies and bees. Here we characterize the contrast sensitivity of motion detection in the praying mantis *Sphodromantis lineola*, an ambush predator that stays stationary for long periods of time while preying on fast-moving prey. In this, its visual behaviour differs from previously studied insects and we might therefore expect its motion detection system to differ from theirs. To investigate the sensitivity of the mantis we analyzed its optomotor response in response to drifting gratings with different contrasts and spatio-temporal frequencies. We find that the contrast sensitivity of the mantis depends on the spatial and temporal frequencies present in the stimulus and is separably tuned to spatial and temporal frequency rather than specifically to object velocity. Our results also suggest that mantises are sensitive to a broad range of velocities, in which they differ from bees and are more similar to hoverflies. We discuss our results in relation to the contrast sensitivities of other insects and the visual ecology of the mantis.

**Electronic supplementary material:**

The online version of this article (doi:10.1007/s00359-015-1008-5) contains supplementary material, which is available to authorized users.

## Introduction

Visual systems need to recognize the features of objects in their environment. Two of the most important visual features that animals need to detect are object movement and the direction of object motion—think of an animal trying to spot a passing mate or a predator trying to capture moving prey. Animals have therefore evolved sophisticated motion detectors that detect both these visual features (Hassenstein and Reichardt 1956). Motion detectors were initially studied and modelled in the visual system of weevils and flies (Hassenstein and Reichardt 1956; Borst and Egelhaaf 1989), but models of motion detectors have been elaborated to be applicable to vertebrates as well, including humans (Adelson and Bergen 1985; van Santen and Sperling 1985; Clifford and Ibbotson 2002). The basic structure of these models comprises a visual sensor whose output is combined in a non-linear interaction (such as a multiplication) with the delayed output of an identical but spatially separated sensor (van Santen and Sperling 1985; Borst and Egelhaaf 1989). This non-linear interaction ensures that the greatest combined response occurs when an object passes first one sensor and then the other (Borst and Egelhaaf 1989). It also ensures that such a system is directionally sensitive, thus enabling visual systems to detect both motion and the direction of movement (Borst and Egelhaaf 1989).

One of the better studied characteristics of the motion detection system in insects is its sensitivity to contrast (Dvorak et al. 1980; Srinivasan and Dvorak 1980). Our knowledge of the contrast sensitivity functions of insects comes largely from neurophysiological studies. There have, however, also been a few behavioural studies that have made use of the optomotor response of flies to investigate motion detection and contrast sensitivity (Reichardt and Wenking 1969; Pick and Buchner 1979; Reichardt and Guo 1986). This is a turning response made by insects in response to free field movement of the background—typically a moving grating in experiments. When presented with a moving background, insects turn in the direction of movement, thus indicating their detected direction of movement (Reichardt and Wenking 1969; Kaiser and Liske 1974; Poggio and Reichardt 1976). This response can be used to investigate at what contrasts motion can be detected and elicits a behavioural response. Evidence from both neurophysiology and behavioural studies show that an insect’s response to a moving background depends on the contrast of the background and that this contrast sensitivity is dependent on both the spatial and the temporal frequency of the background (Reichardt and Wenking 1969; Pick and Buchner 1979; Srinivasan and Dvorak 1980; Reichardt and Guo 1986). The dependency on spatial and temporal frequency could limit the motion detection system’s capability to independently calculate velocity—the visual system would as a result be tuned to the spatio-temporal features of stimulus rather than velocity per se (Hausen and Egelhaaf 1989; but see Straw et al. 2008).

Insects like flies, butterflies and bumblebees have therefore optimized their visual nervous systems to be sensitive to combinations of spatial and temporal frequencies that represent higher velocities (O’Carroll et al. 1996). This matches their fast-moving behaviour where most objects move at a high speed relative to them. Certain insects, however, have alternative strategies that allow them to be sensitive to lower velocities as well (O’Carroll et al. 1996, 1997). Hoverflies and hawkmoths, for example, are sensitive to both higher and lower velocities. This matches their behavioural ecology—they stay hovering stationary in one place (and experience objects moving at low velocities) but also make quick flights (and experience objects moving at high velocities) (O’Carroll et al. 1996). These differences highlight the fact that different mechanisms might underlie the same behaviour in different insects. Furthermore, investigating and generating computational models of the same behaviour in different species could yield important information on commonalities and differences in the mechanisms of motion detection in different insects.

We investigated the contrast sensitivity function in a novel system—the praying mantis *Sphodromantis lineola*. The praying mantis is a predator that tracks and strikes at specific prey types based on several cues including motion (Prete et al. 1999). Motion detection is thus a fundamental cue that enables the sophisticated level of predation we see in the praying mantis. Yet, while there is a wealth of information about visual stimuli that elicit mantis predatory and range-finding behaviour (Rossel 1983; Prete and Mahaffey 1993; Poteser et al. 1995; Prete et al. 2002), little is known about the mechanisms underlying motion detection in this insect (Liske 1999). Of particular interest is how the contrast sensitivity depends on the spatial and temporal frequencies of the background. Since the mantis, like the hoverfly, is largely stationary, one might expect it to be more sensitive to spatio-temporal combinations that allow it to detect low velocities. However, given that it preys on fast-moving insects, it would also need to be able to detect high velocities. In addition to these concerns, a more general motivation for our study is that we lack detailed behavioural data on motion detection and contrast sensitivity from most insects apart from flies. Even in mantises, the optomotor response has remained largely uncharacterized. Our study provides the first detailed characterization of this response and its contrast sensitivity in mantises and investigates how the dependency of this sensitivity on the spatio-temporal frequencies of the background matches the behavioural ecology of the mantis.

## Materials and methods

### Animals

All experiments were carried out on individuals of the species *Sphodromantis lineola*. Six adult females were used in the experiments. All animals were housed in individual plastic boxes (17 cm length × 17 cm breadth × 19 cm height) with holes in the lids to allow for ventilation. The insects were free to move within the boxes. The boxes were stored in an insect housing facility where temperature was maintained at 25 °C. The boxes were cleaned and misted with water twice a week and each individual was fed with a live adult cricket twice a week.

### Experimental setup

**Fig. 1:**
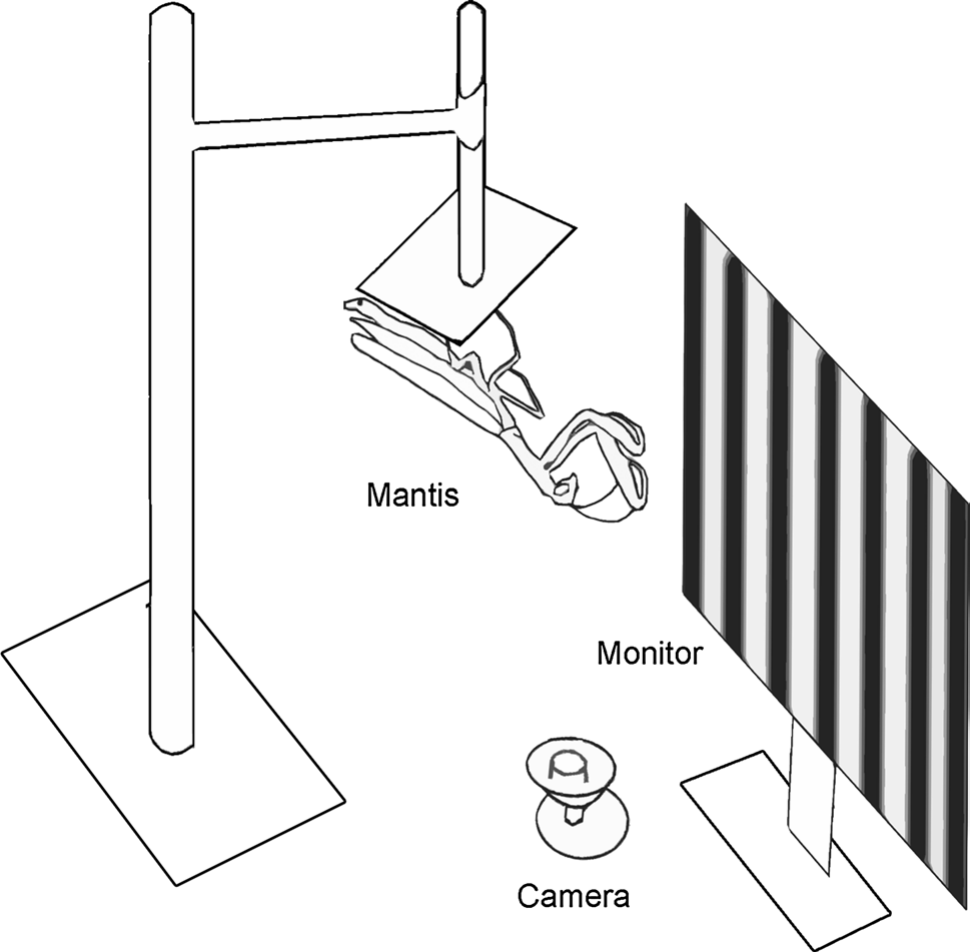
Schematic diagram of the experimental setup. The distance between the mantis’ head and the monitor was 7 cm

For the experiment, animals were allowed to hold on upside down to the square base of a Perspex^®^ holder (length: 14.5 cm; base side: 5 cm) (Fig. 1). There was no constraint on the animals and they were free to move. The stem of the Perspex^®^ holder was held in a clamp that fixed the position of the holder and the mantis. The clamp and holder were positioned so that the mantis was 7 cm in front of a Cathode Ray Tube screen (Hewlett-Packard 21″ colour monitor P1130) with a refresh rate of 85 Hz. The monitor was gamma-corrected and had a spatial resolution of 40 pixels/cm. The screen was 50 cm in width by 32 cm in height and this covered the majority of the visual field of the mantis, subtending a visual angle of 148.7° in width. The entire setup was placed in a custom-built wooden box to prevent visual distraction by movements in the experimental room. The room lights were dimmed during the experiments so that the only light reaching the mantis came from the CRT screen.

A Kinobo USB B3 HD Webcam (Point Set Digital Ltd, Edinburgh, Scotland) was placed directly beneath the mantis and the output of the camera was fed to a DELL Optiplex 9010 computer. The output of the camera was used to monitor the movements of the mantis during the experiment for classification of the mantis’ movements and to ensure the smooth progression of the experiment. The camera was positioned so that the observer only had a view of the mantis and not of the computer screen. The output of the cameras could thus be monitored and coded blind to the condition of the experimental run.

### Visual stimulation

Stimuli were created with a custom written script using Matlab (MathWorks) and the Psychophysics Toolbox (Brainard 1997; Pelli 1997; Kleiner et al. 2007). Every test began with a centring stimulus consisting of a black fly-shaped stimulus against a chequered background. The stimulus spiralled in from the edges of the screen to the centre of the screen immediately in front of the mantis and served to attract the mantis and centre its gaze. Further alignment was made by moving the entire background image to the left or the right and triggering the optomotor response until the mantis was aligned to the centre of the screen (Supplementary Video S1). Once the mantis was aligned, it was presented with the test stimulus. This consisted of a sinusoidal luminance grating filling the entire screen that moved towards either the right or the left for 5 s. In each trial of the experiment, the grating could vary in three characteristics: contrast, temporal frequency and spatial frequency. Across all mantises, trials were run at six different contrast levels for four different spatial frequencies and three different temporal frequencies. The contrast level was defined as Michelson contrast, i.e., the amplitude of the luminance grating divided by its mean luminance. The mean luminance was always 13.18 cd/m^2^, which was half the maximum displayable luminance (“white”). Contrast levels used were 1, 0.5, 0.25, 0.125, 0.0625, 0.03125 and 0.015625. The spatial periods of the gratings were 1600, 400, 100 and 50 pixels. These were converted to spatial frequencies with the following formula—

$${\text{Spatial~frequency}} = \left( {2 \times \arctan \left( {\frac{{{\text{Spatial~period~}}\left( {{\text{px}}} \right)}}{{2 \times {\text{monitor~resolution~}}\left( {\frac{{{\text{px}}}}{{{\text{cm}}}}} \right) \times {\text{viewing~distance}}}}} \right)} \right)^{{ - 1}}$$

The resulting spatial frequencies were approximately 0.0071, 0.0141, 0.0494 and 0.0980 cycles/deg. The temporal frequencies used were 0.25, 8 and 30 cycles per second (Hz). An additional set of trials was later run with gratings of temporal frequency 8 Hz to sample additional spatial frequencies. The contrast values remained the same for this run while the spatial frequencies used were 0.0071, 0.0091, 0.0141, 0.0254, 0.0494, 0.0733, 0.0980, 0.1956 and 0.4073 cycles/deg. The order of presentation of each combination of contrast, spatial frequency and temporal frequency was randomised across an entire experiment. Each trial was presented for 5 s.

These full-screen trial stimuli typically elicited movements of the mantis’ entire body in the direction of the stimulus movement (Supplementary Material Videos S2 and S3). These movements were quite different from the saccadic and tracking responses, generated primarily by movement of the head, which we observe in response to small isolated stimuli (Prete and Mahaffey 1993). These whole-body movements elicited by full-screen stimuli resemble the optomotor response in other species (Reichardt and Wenking 1969; Pick and Buchner 1979; Reichardt and Guo 1986), and we therefore consider them to be the mantis optomotor response, aimed at maintaining postural stability relative to the environment.

In general, insect optomotor responses can vary in their properties (e.g., torque response) as a function of the stimulus properties (e.g., Reichardt and Guo 1986). However, to define a psychophysical contrast threshold, we only need to determine whether an optomotor response was elicited or not. In signal detection theory, eliciting a response corresponds to successfully detecting the signal. We can then define the threshold as being the contrast where the signal is detected on half of trials.

To assess whether an optomotor response was elicited on each trial, the experimenter coded whether the mantis turned left or right, or failed to respond. Subsequently, the mantis was presented with the centring stimulus once more before the next trial was started. A single run of the experiment presented the mantis with one combination each of every spatial frequency, temporal frequency and contrast level. Multiple runs of the experiment were conducted over several days to obtain replicate sampling points from each mantis. Each mantis on average faced 2304 (±1139) trials in total across 9 (±2 SD) runs. All parameters for every trial were saved for later comparison with the analysis of mantis motion in response to the stimulus.

During trials, any movements of the mantis away from the viewing position were noted and these trials were discarded. The mantis was then placed back into the viewing position and the experiment was resumed.

### Analysis

During the experimental runs, the trials were coded blind to the stimulus condition and the movement of the mantis (right, left or no response) was noted for each trial. Subsequent to the experiment, trials coded as right or left were compared with the actual direction of motion of the stimulus and coded as being either in or against the direction of motion of the grating. As described in the Results, responses were hardly ever made against the stimulus direction. A movement in the direction of the stimulus was taken as indicating the detection of movement. The mean proportion of responses in the direction of motion was calculated. These were combined to generate psychometric functions showing the probability of eliciting an optomotor response as a function of stimulus contrast. Psychometric functions were generated for every combination of spatial and temporal frequency tested.

For each combination of frequency, the psychometric data were fitted with a sigmoid (erf) function in the form:

$$y = 0. 5 { } \times \, \left( { 1 + {\text{erf}}\left( {{ \ln }\left( {{\text{contrast}}/{\text{cth}}} \right)/{\text{sqrt}}\left( 2\right)/{\text{sigma}}} \right)} \right)$$

where cth is the contrast detection threshold and sigma is a parameter specifying the steepness of the curve.

We used a least-squares method to fit the sigmoid function for each spatial and temporal frequency pair. An unconstrained linear optimization function (fm in search in Matlab) was used to calculate cth and sigma such that the sum of residual fitting errors squared expressed by

$$S = \mathop \sum \limits_{i = 1}^{N} \left( {y_{i} - p_{i} } \right)^{2}$$

was minimized (where *N* is the number of contrast levels, *y* is the fitted sigmoid function and *p* is the proportion of trials in which the mantises peered in the direction of the stimulus).

The contrast level at which the curve reached 50 % response probability was defined as the threshold contrast level. Contrast sensitivity was defined as the inverse of this threshold value. For two of the curves, the mantises never reached 50 % response probability even at the highest possible contrast of 1. For these, the notional contrast threshold was defined to be where the fitted sigmoid function would cross 50 %, resulting in a sensitivity lower than 1.

The relationship between contrast sensitivity and spatial frequency was then investigated by plotting how sensitivity varied with increasing spatial frequency at each of the three temporal frequencies measured. Similarly the dependency of contrast sensitivity on temporal frequency was investigated by plotting curves of contrast sensitivity against increasing temporal frequency for each spatial frequency used in the experiments. Finally, we fitted a spline surface on the log frequency coordinates using the Matlab (Mathworks) function interp2 and used the fitted surface to generate a contour plot, using the Matlab (Mathworks) contour function. The contour plot plotted contrast sensitivity across the different temporal and spatial frequencies used in the experiment. This plot is especially useful to investigate how combinations of spatial–temporal frequencies restrict movement perception for an organism and therefore what range of velocities is perceivable.

### Correction for observer bias

Data were collected and coded by three different observers but one observer coded the data with consistently lower thresholds. To combine both sets of data, we therefore followed a two-step approach. First, data from the third observer were not used for spatial frequencies at which data were available from the other observers. Second, for spatial frequencies at which data were only available from this observer, data points were corrected for observer bias by dividing the sensitivity values obtained by this observer by a scaling factor. The scaling factor was calculated using spatial frequencies sampled by all observers and chosen to minimize the sum of the squared differences between the sensitivities calculated by the third observer and the average sensitivity calculated by the other observers. These scaled data points are indicated with separate symbols in the curve obtained (triangles in Fig. 3).

## Results

### Psychometric contrast functions

**Fig. 2:**
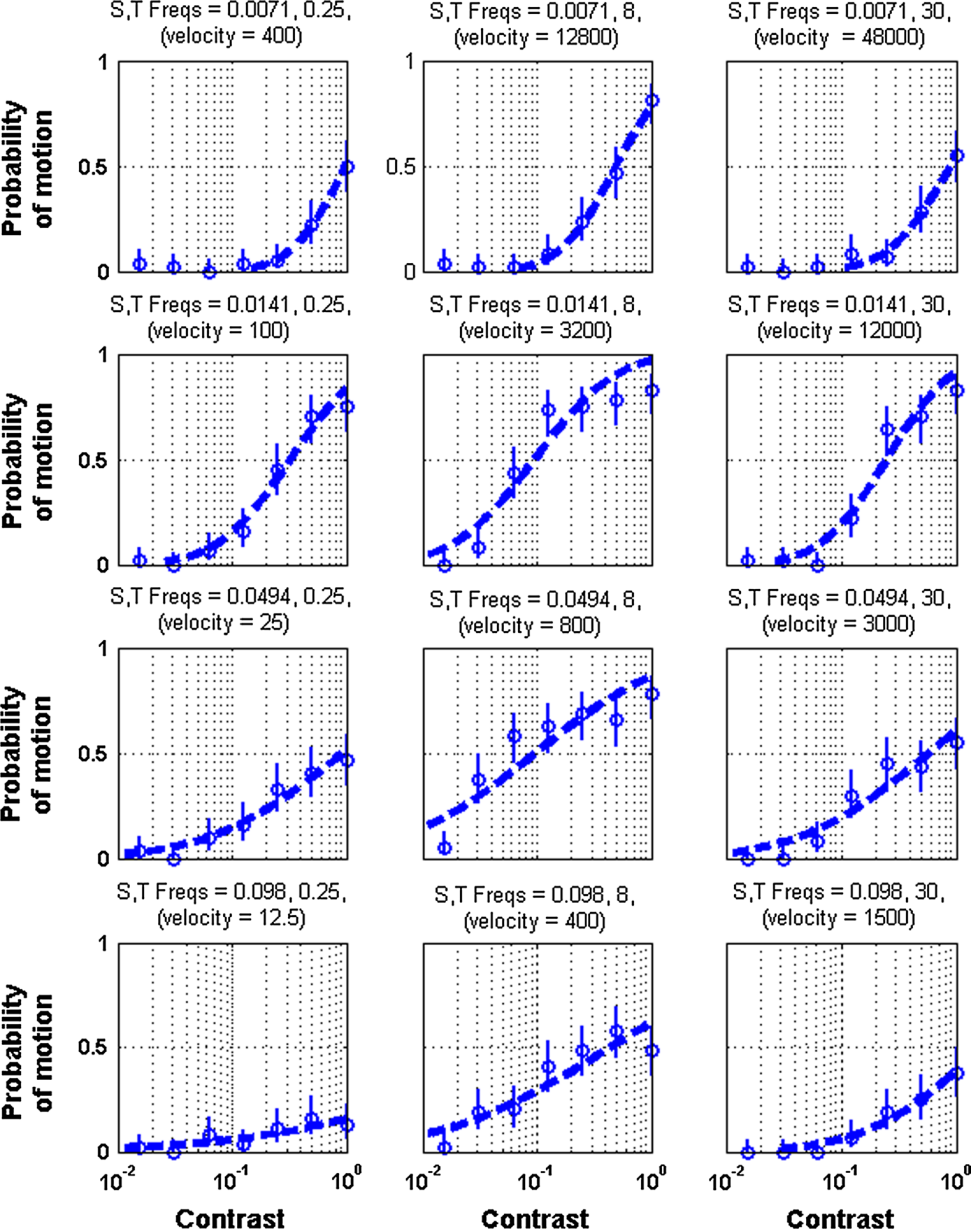
Psychometric functions of the mantis’ optomotor response to gratings of different contrast ratios and varying temporal and spatial frequencies. The spatial (cycles/deg) and temporal frequencies (Hz) are indicated above each plot along with the corresponding velocity (°/s). *Circles* indicate mean (±95 % confidence intervals) number of responses and the *dashed lines* indicate the fitted *curves*. Contrast thresholds were defined as the contrast ratio that elicited a 50 % probability of response. Data are pooled across individuals

In the overwhelming majority of trials (99.4 %), mantises either moved in the direction of motion or showed no response at all. For every spatio-temporal frequency, the probability of motion was a monotonically increasing function of contrast (Fig. 2). In some, but not all, cases this increased until it reached a maximum value within the range of contrasts we tested, after which greater contrast did not increase the probability of response. The steepness or flatness of curve differed with different spatial and temporal frequencies.

### Response to temporal and spatial frequency

**Fig. 3:**
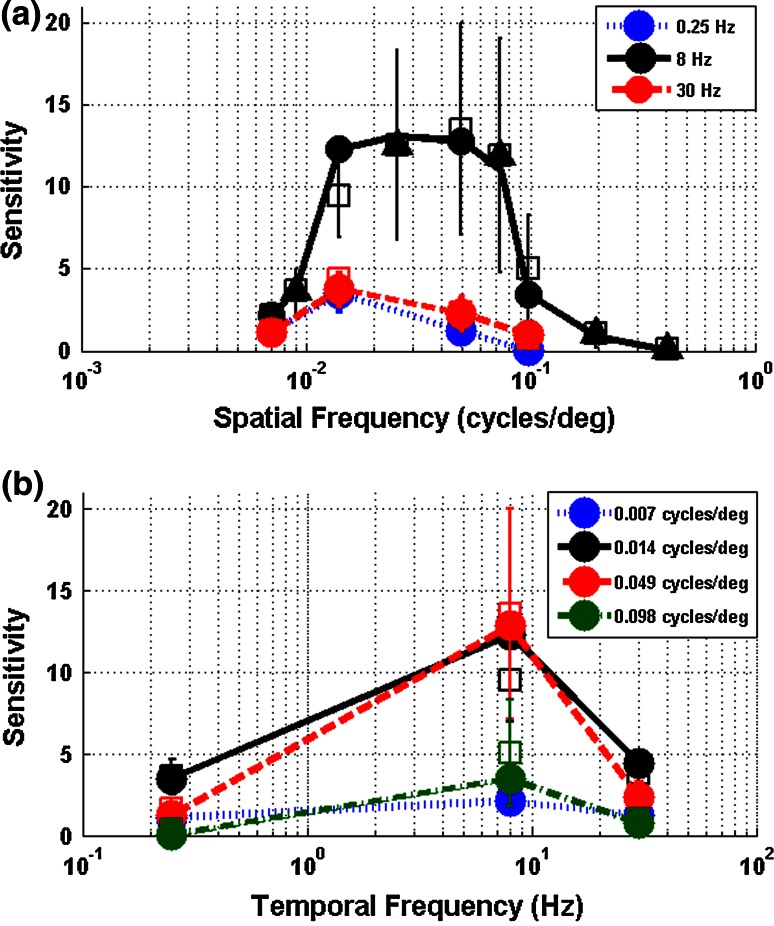
Sensitivity of the mantis’ optomotor response. Mean sensitivity at (**a**) different temporal frequencies across varying spatial frequencies and (**b**) different spatial frequencies across varying temporal frequencies. *Triangles* in the* upper plot* represent data points corrected for observer bias (see main text for further details). *Circles* represent data pooled across all individuals. *Squares* represent means of individual sensitivities and *error bars* represent standard error around these means

To investigate the effect of spatial frequency and temporal frequency in detail we obtained the threshold contrast, defined as the contrast at which the fitted curves plotted in Fig. 2 reached a 50 % response probability. We examined plots of the contrast sensitivity (the reciprocal of the threshold) with respect to varying temporal and spatial frequencies (Fig. 3). Increasing spatial frequency led to an initial increase in the response sensitivity until a peak at around 0.05 cycles/deg, after which there was a decrease in sensitivity (Fig. 3a). This trend seemed to hold for every temporal frequency we tested; the peak sensitivity was, however, different for different temporal frequencies and had the highest peak for a temporal frequency of 8 Hz. The mantises were thus most responsive to a particular combination of spatio-temporal frequencies. This was also borne out through an examination of the relationship between contrast sensitivity and temporal frequency (Fig. 3b). At all four spatial frequencies tested, the contrast sensitivity was higher for 8 Hz than for 0.25 or 30 Hz. This pattern was seen across different spatial frequencies with the highest response peak at a spatial frequency of 0.049 cycles/deg.

### Velocity sensitivity

Sensitivity to different combinations of spatio-temporal frequencies would render animals sensitive to different velocities in their environment. For example, higher sensitivity to a combination of high temporal velocities only at lower spatial frequencies would help an animal detect objects moving at higher velocities, while sensitivity to higher spatial frequencies only at low temporal frequencies would help the animal detect objects moving at lower velocities. Additionally, we can ask whether the mantis response is tuned to velocity per se, or whether it is tuned separably to spatial and temporal frequency. If it is tuned to velocity *V*_pref_, then at a given temporal frequency $\nu$ the mantis will respond best to the spatial frequency *f* for which *f* = $\nu/ V_{pref}$. Conversely, if it is tuned to spatial frequency *f*_pref_, then it will always respond best when *f* = *f*_pref_, irrespective of temporal frequency or velocity. We asked whether the mantis behavioural contrast sensitivity function was best described as a function of velocity, or as a separable function of spatial and temporal frequency.

**Fig. 4:**
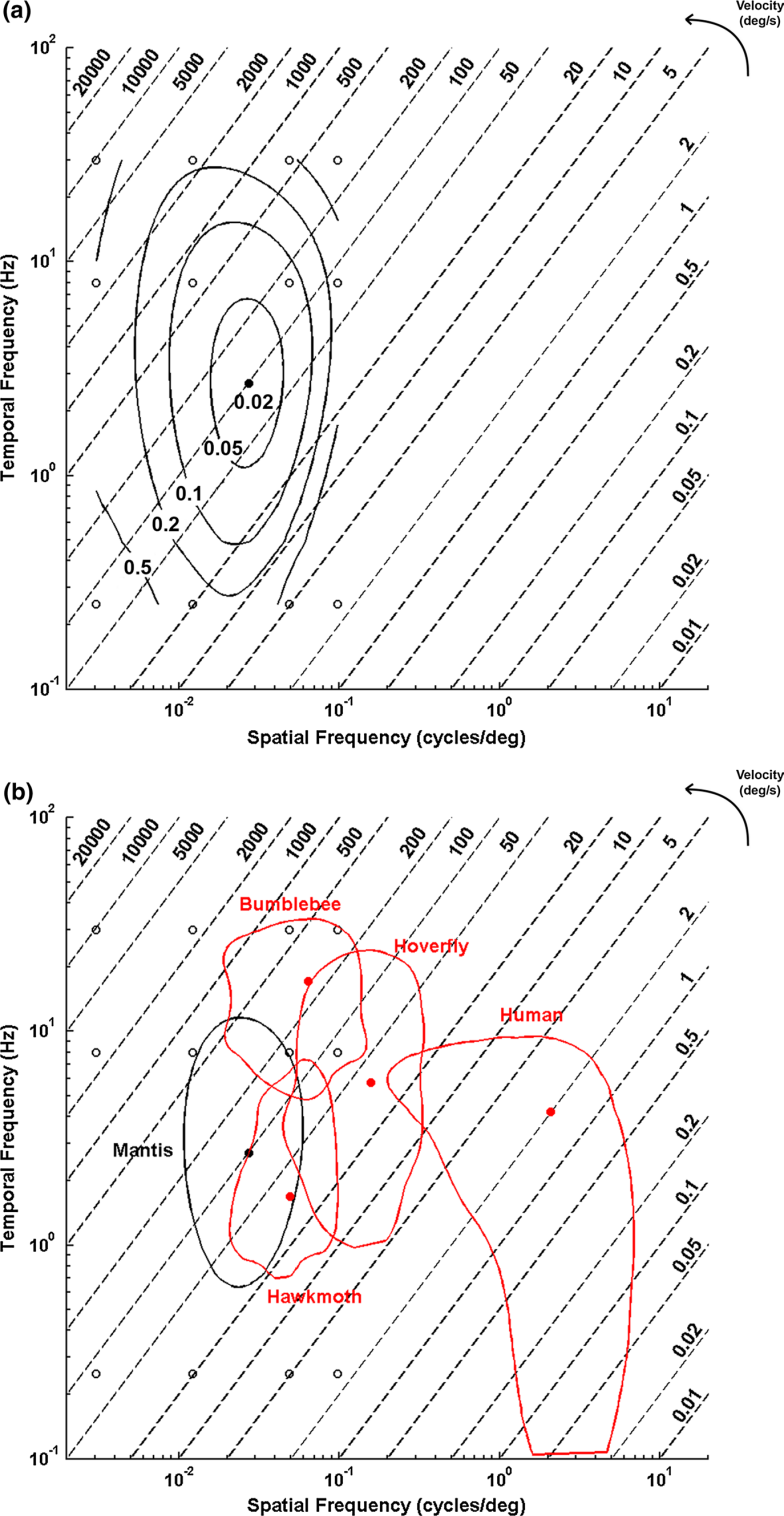
Contour plots of contrast thresholds.* Contour lines* represent (**a**) isolines of mantis contrast thresholds across different spatial and temporal frequencies and (**b**) the locus where contrast sensitivity falls to half maximum in the mantis (*black line* and *dot*) compared to other species [*red lines* and *dots*; data after (O’Carroll et al. 1996)]: hawkmoths (*Deilephila*), hoverflies (*Volucella*), bumblebees (*Bombus*) and humans. Linear interpolation done by Matlab interp2 function. *Diagonal dashed lines* indicate velocities. *Filled central dots* represent regions of maximum sensitivity in all species. *Open circles* represent the data used to interpolate the contour for the mantis. Interpolation indicates our mantises display their optimal sensitivity at a spatial frequency of around 0.03 cycles/deg and a temporal frequency of 2.7 Hz. At this point, their contrast threshold is around 0.038

To this end, we generated a contour plot of the contrast threshold isolines across different combinations of spatial and temporal frequencies (Fig. 4a). This plot revealed that mantis contrast sensitivity is at least roughly a separable function of spatial and temporal frequency: the contours are symmetric with respect to the spatial and temporal frequency axes, and there is little evidence of any tuning specifically to velocity.

The contour fit implies that mantises are most sensitive when the spatial frequency is around 0.03 cycles/deg and the temporal frequency is around 3 Hz. In Fig. 4b, we plot the contour isolines bounding the spatio-temporal frequencies where mantis sensitivity is half of its maximum value, and superimpose similar iso-lines for other species (O’Carroll et al. 1996). Mantises are most sensitive to relatively low spatial frequencies (between 0.01 and 0.1 cycles/deg) compared to humans, but overlap in sensitivity to temporal frequencies (between around 1 and 10 Hz) (Fig. 4b).

## Discussion

We investigated the contrast sensitivity of the mantis *Sphodromantis lineola* using its behavioural response to moving grating stimuli of different spatial and temporal frequencies. Since the stimuli used were wide-field stimuli, covering nearly 150^o^ of visual angle and the movements elicited were movements of the mantis’ entire body, we believe that these movements are best considered as the mantis’ optomotor response: an attempt to use visual feedback to maintain postural stability in the world. Our results are therefore specific to this response; other visual responses such as a possible small-target tracking response might have differing contrast sensitivity.

The optomotor response of the mantis has so far been poorly studied. The single study that has investigated this before examined how several factors including the hunger level of mantises as well as head-grooming and copulatory behaviour influence the optomotor response (Liske 1999), but did not study the effect of different visual stimuli on the optomotor response. Our study shows that the optomotor response in the mantis is a reliable behaviour that can be used to investigate its visual capabilities as has been done in flies.

Our results further show that the response in this mantis species has a contrast sensitivity that varies with spatial and temporal frequency of the stimulus. The sensitivity seems to be separably tuned to temporal and spatial frequency, rather than directly to velocity. It shows bandpass tuning to both spatial and temporal frequency, i.e., sensitivity falls off with either a decrease or increase away from the preferred frequency. In the spatial domain, the decline in sensitivity to high frequencies probably reflects optical limits. The inter-ommatidial angle in the mantis reaches a minimum of around 0.6^o^ at the fovea (Rossel 1979), and is around 2.5 ^o^ in the peripheral areas on which the optomotor response may predominantly depend, corresponding to a Nyquist limit of around 0.8 and 0.2 cycles/deg, respectively. In fair agreement, we found little sensitivity to spatial frequencies above 0.1 cycles/deg. In other species, the decline in sensitivity at low spatial frequencies is due to neuronal mechanisms such as lateral inhibition (Barten 1999). This probably applies to mantises as well. In the temporal domain, the decline in sensitivity to high frequencies probably represents finite integration times within the neuronal mechanisms responsible for motion detection, while the decline at low frequencies probably represents adaptation, i.e., the longest time over which “sustained” neuronal mechanisms maintain their response.

### Spatio-temporal separability

In humans, the limits of visibility are roughly separable in spatial and temporal frequency (Robson 1966; Kelly 1979; Watson et al. 1986). The highest spatial frequency we can resolve remains near-constant over a wide range of temporal frequencies, and the fastest flicker we can perceive remains near-constant over a wide range spatial frequencies. Conversely, the fastest velocity we can perceive is not constant, but depends on frequency: we can perceive rapid motion of low spatial frequency patterns, but only slow motion of high-frequency patterns. The separability of these limits are thought to reflect the properties of neurons in early areas, which are tuned separably to specific spatial and temporal frequencies; tuning to velocity is constructed by later processing of these inputs (Perrone and Thiele 2001; Priebe et al. 2006; Umino et al. 2008).

The limits of visibility for mantises also appear to be roughly separable in spatial and temporal frequency. There is little evidence of tuning for speed, which would appear as contours elongated parallel to the velocity isolines. Mantises show greatest sensitivity to gratings at a spatial frequency of around 0.03 cycles/deg, and their sensitivity falls to half-maximal at around 0.1 cycles/deg for almost all visible temporal frequencies.

### Contrast sensitivity and visual ecology

The contrast sensitivity functions of insects are well matched to their visual ecologies (O’Carroll et al. 1996). Flies and bees, for example, need to be sensitive to stimuli at high velocities due to the speeds at which they fly and their contrast sensitivities are correspondingly tuned to spatial and temporal frequencies that enable this (O’Carroll et al. 1996). It is interesting to see how the tuning to specific spatial and temporal frequencies limits the ability of the mantis to detect velocities. Mantises’ good sensitivity to a range of temporal frequencies (around 0.6–10 Hz) and low spatial frequencies (<0.1 cycles/deg) suit them for perceiving a wide range of speeds. Their optimal sensitivity corresponds to a velocity of 100 deg/s—faster than humans can perceive with half-maximal sensitivity (Fig. 4b). Humans are most sensitive to velocities an order of magnitude lower, around 2 deg/s. The contour bounding mantis half-maximal sensitivity, however, spans a large range of velocities ranging from 500 to close to 20 deg/s (Fig. 4b). Thus, stimuli moving at around 30 deg/s can be perceived with equal sensitivity by humans and mantises, although the two species achieve peak sensitivity to this velocity at different spatial frequencies.

It is important to point out that our study uses behaviour to investigate the contrast sensitivity function, while most previous studies in insects use neurophysiology. The hawkmoth, hoverfly and bumblebee contrast sensitivity in Fig. 4b are derived from neuronal responses, although the human data is behavioural (Kelly 1979). Since we relied on only the behavioural response to generate the contrast sensitivity curves, we cannot comment on whether neurophysiological estimates of the contrast sensitivity in this species would differ. Previous studies comparing optomotor/optokinetic with neuronal measures in other species have found that optomotor/optokinetic estimates of contrast sensitivity tend to be lower overall, but are qualitatively similar with respect to shape and peak sensitivity in response to changes in frequency (e.g., Donaghy 1980). Additionally, the fact that our behavioural measures found the greatest sensitivity at a velocity of 100 deg/s also argues for the general applicability of our results. Such high speeds are presumably more ecologically relevant to prey capture rather than to postural stabilisation. This suggests that our experiment is probing the properties of early neuronal mechanisms which subserve both prey capture and postural stability, making it more likely that similar results would be obtained with other methods. Another important caveat is that our contour plot relies on a much poorer sampling of the spatio-temporal space than the neurophysiological data in other insects (O’Carroll et al. 1996) and as such should be taken as indicative of the range of possibilities rather than definitive limits. With these caveats, we have compared our behavioural data with neurophysiological data from other insect species in Fig. 4b.

In its tuning to a broad range of temporal frequencies and velocities, mantis behavioural contrast sensitivity differs from the visual systems of bees (Fig. 4b) and is similar to the hawkmoth *Deilephila* and the hoverfly *Volucella*, two insects with which the mantis shares some aspects of visual behaviour (O’Carroll et al. 1996; Fig. 4b). Insects such as these hover and thus need contrast sensitivity that also enables them to be responsive to stimuli at low (by insect standards) velocities. Other hovering insects like hummingbird hawkmoths (*Macroglossum*) and hovering bee-flies (*Bombylius*) also have sensitivity functions sensitive to stimuli at lower velocities (O’Carroll et al. 1997). In the hummingbird hawkmoth, this is enabled by a tuning to higher spatial and temporal frequencies, while in the bee-fly, the sensitivity function has two peaks—one at higher temporal frequencies and one at lower temporal frequencies which enables it to be sensitive to both higher and lower velocities (O’Carroll et al. 1997). Like these hovering insects, mantises also spend large periods of time relatively stationary and therefore would presumably experience low object velocities compared to insects like bees for which vision is most relevant when flying. Our results show that mantis’ vision is tuned to lower spatial frequencies than hoverflies and hawkmoths. However, mantis vision—at least as assessed via these behavioural experiments—appears to be similar to theirs in being sensitive to lower velocities than bees. While the tuning to lower velocities reflects what is common in their visual ecology, hawkmoths and hoverflies probably need a tuning to higher velocities for the short flights they make in between periods of hovering (O’Carroll et al. 1997). Since mantises do not make short flights in between their stationary periods, sensitivity to higher velocities probably does not reflect similar adaptation in mantises. It might instead enable mantises to spot fast-moving prey like flies. This combination of staying stationary and capturing fast-moving prey might explain why the visual system in *S.lineola* is tuned to detect higher as well as lower velocities. It would be interesting to see if mantises with different prey-capture strategies differ in the tuning of their contrast sensitivity functions.

In conclusion, the contrast sensitivity of the mantis appears to differ in some aspects from those of primates and fast-flying insects (bees, flies) but shares several characteristics with the contrast sensitivity functions of hovering insects (bee-flies, hawkmoths). The mantis contrast sensitivity function thus probably reflects its visual ecology and specialization as an ambush predator on fast-moving prey.

## Electronic supplementary material

Supplementary Video S1. Exemplar video of the alignment stimulus used to interactively centre the mantis between trials and the response of the mantis. Note that this video was not recorded during an actual experiment but is for representative purposes only. Supplementary material 1 (MPG 2371 kb)

Supplementary Videos S2 and S3. Exemplar videos of the stimulus used in experiments and the corresponding optomotor response of the mantis. Note that this video was not recorded during an actual experiment but is for representative purposes only. Actual videos recorded during experiments were recorded blind and did not have a view of the stimulus. Supplementary material 2 (MPG 2107 kb)

Supplementary material 3 (MPG 1760 kb)
